# Gut health and microbiome modulation by the heat-killed postbiotic beLP1^®^ in adults with excess weight: a randomized, placebo-controlled trial

**DOI:** 10.3389/fnut.2026.1919277

**Published:** 2026-09-17

**Authors:** Ranjith Kumar Manoharan, Kwon-Il Han, Yura Lee, Sunhwa Baek, Eunjung Moon, Youn Bum Park, Junhui Cho, Jayesh Chaudhary, Hyun-Dong Shin

**Affiliations:** 1 Research & Development Center, Bereum Co., Ltd., Wonju, Republic of Korea; 2 Vedic Lifesciences, Mumbai, India

**Keywords:** digestive symptoms, gastrointestinal microbiome, GSRS, gut microbiota, *Lactiplantibacillus plantarum*, quality of life, stress

## Abstract

**Background:**

Overweight is linked to gut microbiota dysbiosis and gastrointestinal (GI) symptoms that impair quality of life.

**Methods:**

This 84-day randomized, double-blind, placebo-controlled study evaluated the gut-health effects of heat-killed *Lactiplantibacillus plantarum* (beLP1^®^) in otherwise healthy adults (aged 18–45 years) within the BMI range of 25–35 kg/m^2^ and a ≥3-month history of mild-to-moderate GI discomfort, confirmed by Gastrointestinal Symptom Rating Scale (GSRS). In this trail 140 adults were allocated in a 1:1 ratio to receive either daily beLP1^®^ (>30 billion cells) (*n* = 70) or placebo (*n* = 70). A total of 103 participants completed the study without protocol deviations and were included in the primary per-protocol (PP) analysis (beLP1^®^: *n* = 49; Placebo: *n* = 54). This predefined analysis directly compares the beLP1^®^ and placebo arms using analysis of covariance (ANCOVA), supplemented by two independent-sample Student’s *t*-tests. The primary endpoint was change in gastrointestinal symptoms via the GSRS, secondary endpoints included the Perceived Stress Scale (PSS), the Digestion-associated Quality of Life Questionnaire (DQLQ), Tumor Necrosis Factor-alpha (TNF-α), lipid parameters, Dual-Energy X-ray Absorptiometry (DEXA), and gut microbiome diversity by metagenomic Next-Generation Sequencing (NGS).

**Results:**

Compared to placebo, beLP1^®^ significantly reduced total Gastrointestinal Symptom Rating Scale (GSRS) scores at Day 42 (*p* = 0.0215) and Day 84 (*p* = 0.0394), with prominent improvements in abdominal pain (*p* = 0.0205) and dyspeptic syndrome (*p* = 0.0303) domains. Significant reductions in perceived stress (*p* = 0.0004) and improvements in digestion-associated quality of life (*p* = 0.0008) were concurrently achieved. Metagenomic analysis revealed a favorable modulation of the gut microbiota, characterized by an enrichment of several short-chain fatty acid-producing bacteria and a significant reduction in multiple opportunistic pathogens. No significant changes occurred in serum TNF-α, lipid profiles, or DEXA body composition metrics.

**Conclusion:**

Overall, beLP1^®^ safely and progressively alleviates GI symptoms, reduces stress, and optimizes microbiome composition in adults with excess weight. ClinicalTrials.gov: NCT05820737.

**Clinical trial registration:**

https://clinicaltrials.gov/study/NCT05820737?cond=NCT05820, identifier NCT05820737.

## 1 Introduction

Growing evidence highlights the gut microbiota as an integral player in human physiology, exhibiting strong observational and functional associations with metabolic homeostasis, immune activity, and gastrointestinal stability. Disruptions in microbial community structure or functional capacity-commonly described as dysbiosis-are frequently observed alongside intestinal and systemic metabolic disorders (1). Individuals with excess weight commonly exhibit alterations in gut microbiota composition and frequently experience GI that adversely affect quality of life. According to the World Health Organization (WHO), approximately 2.5 billion adults were overweight and 890 million were living with obesity worldwide in 2022 (2). Environmental factors, particularly unhealthy eating habits, contribute substantially to dysbiosis in this demographic. Intervening at the overweight stage with functional nutritional strategies presents a critical therapeutic window to restore microbial balance and alleviate functional GI distress before severe, irreversible metabolic sequelae develop. Among emerging strategies, postbiotics have attracted attention for their potential to support metabolic regulation and gut health (3, 4). The International Scientific Association for Probiotics and Prebiotics defines postbiotics as preparations of inanimate microorganisms and/or their components that confer a health benefit on the host. These comprise heat-inactivated whole cells and bioactive constituents such as cell wall fragments, peptidoglycans, short-chain fatty acids (SCFAs), and extracellular polysaccharides that retain biological activity despite microbial inactivation (5, 6). In addition to their ability to beneficially modulate the gut microbiome (7), postbiotics possess superior inherent stability, precise dosing, and ease of handling compared with live probiotics, positioning them as attractive functional ingredients for dietary supplement applications (8).

Among postbiotic candidates, *Lactiplantibacillus plantarum* species have been widely investigated for their gut health-promoting properties. Certain *L. plantarum* strains enhance intestinal mucin production, strengthen epithelial barrier integrity, and exert anti-inflammatory effects by suppressing pro-inflammatory cytokine cascades, including interleukin-6 (IL-6) and tumor necrosis factor-α (TNF-α) (9, 10). Clinical trials evaluating heat-killed *L. plantarum* strains (such as TWK10) have demonstrated improvements in gut microbiota composition and physical fatigue, supporting the functional efficacy of inactivated *L. plantarum* preparations (11, 12). Although substantial *in vitro* and preclinical evidence supports the beneficial effects of probiotics, prebiotics, and postbiotics, clinical evidence demonstrating their impact on human gut metabolism remains comparatively limited. These microbiome-targeted interventions have also emerged as complementary approaches to conventional antibiotic therapy by supporting microbial resilience and potentially reducing reliance on antibiotics, thereby contributing to efforts to address antimicrobial resistance. Nevertheless, further well-designed clinical studies are required to establish their therapeutic relevance and clinical benefits in humans (13). However, health benefits derived from postbiotics are strictly strain-specific, necessitating rigorous evaluation of individual candidates. The postbiotic strain, *L. plantarum* beLP1^®^ originally isolated from traditional fermented kimchi, has demonstrated complete genomic safety lacking transferable antibiotic resistance genes (ARGs) as well as potent *in vitro* antimicrobial activity against gut pathogens (14). Preclinically, heat-killed beLP1^®^ supplementation in a high-fat diet mouse model attenuated metabolic strain, reduced hepatic stress markers, and favorably modulated gut microbiota composition and SCFA production (15). Furthermore, *L. plantarum* species are widely recognized as safe for human consumption and are extensively utilized in fermented foods, dietary supplements, and functional food products, supporting their translational and commercial applicability (16). Despite these promising preclinical findings, human clinical trials establishing the therapeutic efficacy of heat-killed beLP1^®^ in individuals with excess weight and gastrointestinal distress have not been reported.

Accordingly, the present randomized, double-blind, placebo-controlled trial evaluated the clinical efficacy, safety, and microbiome-modulating effects of daily heat-killed beLP1^®^ supplementation over 84 days in otherwise healthy adults with excess weight and self-reported mild-to-moderate GI discomfort. Primary and secondary endpoints evaluated changes in gastrointestinal symptom severity, perceived stress, digestion-associated quality of life, systemic inflammatory markers (TNF-α), lipid profiles, DEXA body composition, and gut microbiota taxonomic and resistome composition via shotgun metagenomic sequencing.

## 2 Materials and methods

### 2.1 Compliance with ethical standards

The trial was performed in accordance with the Declaration of Helsinki (Ethical Principles for Medical Research Involving Human Subjects, revised by the World Medical Association General Assembly, Seoul, 2008), the International Council for Harmonization (ICH) Guideline for Good Clinical Practice E6 (R2), 2016, and the National Ethical Guidelines for Biomedical and Health Research Involving Human Participants (2017) issued by the Indian Council of Medical Research (ICMR). The protocol was reviewed and approved by multiple ethics committees, namely, Muktai Hospital, Altezza Institutional Ethics Committee, Supreme Independent Ethics Committee, Jivanrekha Institutional Ethics Committee, and Leelavati Ethics Committee and Health Care. The study was registered with ClinicalTrials.gov (Identifier - NCT05820737) and the Clinical Trials Registry of India (CTRI/2023/05/052961).

### 2.2 Study participants

The trial was carried out across eight clinical centers in Maharashtra, India, from April 2024 to March 2025. Potential participants were systematically screened against pre-specified eligibility criteria prior to enrolment. Eligible individuals otherwise healthy adults (aged 18–45 years) within the BMI range of 25–35 kg/m^2^ who maintained an active lifestyle and fulfilled all of the following conditions: a moderate level of physical activity, as determined by the International Physical Activity Questionnaire-Short Form (IPAQ-SF); a documented history of mild to moderate gastrointestinal discomfort persisting for at least 3 months, accompanied by prior use of prescribed medication for symptom relief; and moderate-severity gastrointestinal symptoms, defined by a combined score of 15–29 on the dyspeptic syndrome and bowel dysfunction subscales of the Gastrointestinal Symptom Rating Scale (GSRS) over the preceding 2 weeks. A comprehensive description of all inclusion and exclusion criteria is provided in Supplementary Table 1. Prior to performing any screening procedures or eligibility assessments, written informed consent was voluntarily provided and signed by each participant in accordance with ICH-GCP guidelines. Following a 2-week run-in period, participants were randomized provided they met predefined criteria, including stable body weight (≤2 kg variation), GSRS scores not below screening values (with the highest score recorded during the run-in period considered), submission of both weekly at-home GSRS diaries, ≥80% compliance with the run-in medication, and documentation of any gastrointestinal-related concomitant therapies.

### 2.3 Study design and product

This was a randomized, double-blind, placebo-controlled, parallel-group study in which participants and study investigators remained blinded to treatment allocation throughout the study. Participants were assigned in a 1:1:1 ratio using stratified block randomization (block size of six; StatsDirect v3.1.17) to receive either, beLP1^®^, EF-2001^®^ or placebo; however, the present manuscript reports outcomes for the beLP1^®^ and placebo arms only. At screening, written informed consent was obtained, and participants completed exercise history documentation, the IPAQ-SF, GSRS, and baseline laboratory evaluations, followed by a 2-week run-in period. At randomization, demographic details, baseline characteristics, vital signs, clinical examination findings, medical and medication history, and concomitant medication use were recorded, and the GSRS, PSS, and DQLQ were administered. Blood and fecal samples were collected for safety laboratory testing, metagenomic NGS, and SCFA analyses. Serum TNF-α and lipid parameters were measured using commercial kits (Diaclone; Roche Cobas). Stool samples obtained at baseline and Day 84 underwent shotgun metagenomic sequencing on an Illumina NovaSeq platform, with subsequent taxonomic, functional, microbial diversity (Shannon and Bray-Curtis), and antibiotic resistance gene analyses conducted using established pipelines with false discovery rate correction.

The current study involved oral administration of either 1 capsule of beLP1^®^ or a placebo daily after breakfast for 84 days. Bereum, Republic of Korea, manufactured the capsules where each beLP1^®^ capsule contained ∼10 mg (≥30 billion cells)/day of the heat-killed (beLP1^®^) and 390 mg of Microcrystalline cellulose (MCC), with the total content of the capsule to be 400 mg while the placebo capsule contained 400 mg of MCC. Blinding was maintained by ensuring that the investigational product and placebo were identical in size, shape, color, and texture. Participants who achieved at least 90% compliance during the intervention period were considered compliant and included in the per-protocol (PP) population.

### 2.4 Primary and secondary outcomes

Participants completed study questionnaires at baseline (Day 0), Day 42, and Day 84. These questionnaires captured gastrointestinal symptom severity and domain scores, perceived stress, digestion-related quality of life, treatment adherence, concomitant medication use, and any adverse events. The primary endpoint was the change in total GSRS scores. The GSRS is a validated self-report instrument comprising 18 items rated on a 4-point Likert scale, with higher scores indicating greater symptom severity. It assesses four symptom domains: abdominal pain, dyspepsia, indigestion, and bowel dysfunction. Domain-specific and overall scores were derived as the means of the relevant item responses (17, 18).

Secondary endpoints included changes in perceived stress measured by the PSS, a widely used and validated instrument for evaluating perceived stress and emotional responses to daily situations. In this study, improvement in stress was defined as a reduction in PSS scores after 84 days of intervention (19). Digestion-related quality of life was evaluated using the validated DQLQ, a nine-item questionnaire assessing how digestive symptoms affected physical and psychological aspects of daily life over the previous week. Total DQLQ scores ranged from 0 to 9, with higher scores indicating poorer digestion-related quality of life (20).

Inflammatory status was assessed by measuring serum TNF-α, a key pro-inflammatory cytokine and adipokine implicated in chronic low-grade inflammation associated with overweight and obesity. Serum TNF-α concentrations were measured at baseline and at study completion using a validated immunoassay, and changes from baseline were analyzed to evaluate intervention-related effects on systemic inflammation (21–23). Fasting lipid parameters were analyzed to explore potential effects on cardiometabolic risk. Individuals with increased adiposity often exhibit lipid abnormalities, including elevated triglycerides, reduced HDL cholesterol, and altered total and LDL cholesterol levels, which are associated with heightened cardiovascular risk (24). Whole-body composition and fat distribution were measured using DEXA, a precise and validated technique comparable to hydrostatic weighing. DEXA-derived indices were used to examine potential changes in fat mass and lean mass, parameters that are closely related to gastrointestinal health, nutritional status, and metabolic function (24).

Gut microbiota composition and diversity were evaluated through NGS-based analysis of fecal samples collected before and after the intervention. This approach enabled detailed profiling of microbial taxa and diversity metrics to assess potential modulation of microbiota-related metabolic pathways, given the established role of the gut microbiome in energy balance and metabolic disorders (25).

Safety was monitored throughout the study through periodic assessment of vital signs, including pulse rate and blood pressure, and continuous surveillance for adverse events.

### 2.5 Compliance and adverse event reporting

Compliance was assessed by monitoring the number of capsules dispensed and returned, using bottle counts. Concomitant medications and adverse events were also monitored throughout the study.

### 2.6 Statistical analysis

Continuous variables were summarized as mean and standard deviation (SD), while categorical variables were presented as counts and percentages. Data normality was evaluated using the Shapiro-Wilk test supplemented by graphical inspection (histograms and box plots). Between-group baseline demographic and clinical characteristics were compared using two-sample Student’s *t*-tests for continuous variables and Chi-square or Fisher’s exact tests for categorical variables. Primary and secondary between-group efficacy outcomes including questionnaire summary scores (GSRS, PSS, DQLQ) and continuous laboratory parameters were evaluated at post-baseline visits (Day 42 and Day 84 independently) using one-way Analysis of Covariance (ANCOVA) models with Treatment Group (beLP1^®^ vs. Placebo) as the main factor and baseline value as the covariate, alongside two independent-sample Student’s *t*-tests. Within-group changes from baseline to post-baseline visits were evaluated using paired Student’s *t*-tests. All statistical hypothesis tests were two-tailed with a significance threshold set at *p* < 0.05. Statistical analyses were performed on the Per-Protocol (PP) population (*n* = 49 in beLP1^®^; *n* = 54 in Placebo), which included participants who completed all study visits without major protocol deviations or intake of prohibited concomitant medications.

### 2.7 Sample size calculation

The target sample size of 70 participants per arm was determined based on evidence from a published meta-analysis and randomized clinical trials evaluating the clinical effects of *L. plantarum* supplementation, while also considering the exploratory nature of the present study (25). Sample size was calculated using G*Power software (v3.1.9.7) (26) based on detecting a clinically meaningful between-group difference in total GSRS score reductions at Day 84. Assuming a medium effect size (Cohen’s *d* = 0.50), a standard deviation of 5.5 points on the GSRS scale, a two-tailed significance level (α = 0.05), and 80% statistical power (1−β = 0.80), a minimum of 63 evaluable participants per group was required. To account for an anticipated drop-out/attrition rate of approximately 10%–12%, the target allocation was set to 70 participants per arm (210 total across the 3 parent trial arms). Of 357 individuals screened, 209 eligible participants were randomized in a 1:1:1 ratio across three groups. For the primary two-arm analysis comparing beLP1^®^ and placebo, a total of 140 participants were allocated (70 per group). In the beLP1^®^ arm, 51 participants completed the intervention and 19 withdrew or were lost to follow-up, resulting in a PP population of *n* = 49 and a Full Analysis Set (FAS) of *n* = 54. In the placebo arm, 54 participants completed the intervention and 16 withdrew or were lost to follow-up, resulting in a PP population of *n* = 54 and an FAS of *n* = 62. Detailed participant flow and disposition across all stages are presented in Figure 1.

**FIGURE 1:**
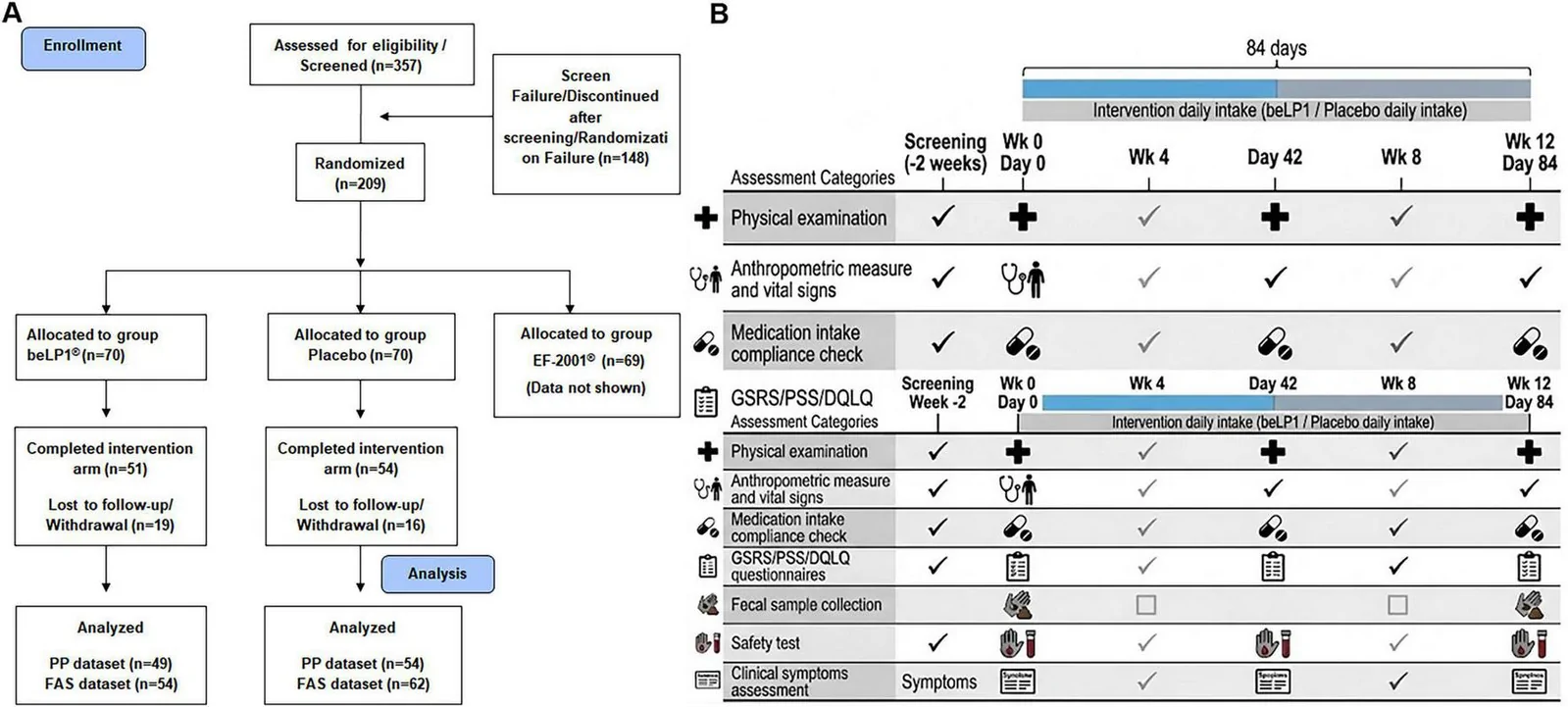
CONSORT study flow diagram and experimental timeline. **(A)** CONSORT flow chart tracking participant progression. Out of 209 met eligibility criteria and were randomized (1:1:1) into the active intervention group (beLP1; *n* = 70) or the control group (Placebo; *n* = 70). After accounting for step-wise discontinuations, the final per-protocol analysis included *n* = 49 in the beLP1 group and *n* = 54 in the Placebo group. **(B)** Schematic diagram of the clinical trial timeline. The randomized, double-blind, placebo-controlled study spans a 2-week screening window followed by an 84-day (12-week) intervention phase. Safety, compliance, clinical score evaluations (GSRS, PSS, DQLQ), and fecal sample collections for microbiota profiling were performed at baseline (Day 0), mid-point (Day 42), and study endpoint (Day 84).

### 2.8 Microbiome DNA extraction, sequencing and sequencing data preprocessing

Next-Generation Sequencing analyses of fecal samples were performed by Cmbio (Copenhagen, Denmark), including DNA extraction, shotgun metagenomic, and sequencing data preprocessing. Fecal samples (∼0.1 g) were subjected to genomic DNA extraction using a commercially available DNA isolation kit (Macherey-Nagel), incorporating mechanical lysis via bead beating to ensure efficient recovery of microbial DNA. Negative (sterile water) and positive (mock microbial community standard, ZymoBIOMICS) controls were included in each batch and processed through all downstream steps to ensure quality assurance.

DNA libraries were prepared using validated library preparation kits (Watchmaker Biosciences and Twist Bioscience) involving enzymatic fragmentation, adapter ligation, and PCR amplification (16 cycles), followed by purification using AMPure magnetic beads (Beckman Coulter, Brea, CA). Library concentration and quality were assessed using fluorometric methods (Qubit dsDNA BR Assay, Thermo Fisher Scientific, Waltham, MA) and fragment analysis prior to sequencing. Shotgun metagenomic sequencing was performed on an Illumina NovaSeq platform with paired-end 150 bp reads at Cmbio (Copenhagen, Denmark), while subsequent bioinformatic analyses were conducted using their established pipelines.

Raw sequencing reads were quality-filtered to remove adapter sequences and low-quality bases (Phred score < 30), and host-derived reads were excluded by aligning to the human reference genome (GRCh38) using established algorithms. Taxonomic profiling and relative abundance estimation were performed using validated bioinformatic pipelines (e.g., CHAMP), with rarefaction applied to standardize sequencing depth across samples. Functional annotation of microbial genes was performed against the KEGG Orthology database, with pathway-level inference based on module-completeness criteria. Antibiotic resistance genes were identified using curated databases (e.g., CARD) with stringent filtering (perfect and strict hits), and relative abundance profiles were generated accordingly.

## 3 Results

### 3.1 CONSORT flow chart and baseline characteristics of study participants

Participants in the beLP1^®^ and placebo groups were comparable at baseline. Supplementary Table 2 summarizes the participants’ demographics and baseline characteristics. Overall, 103 participants, composed of beLP1^®^ (*n* = 49) and placebo (*n* = 54), met the criteria for inclusion in the PP population, i.e., the participants without any major protocol violations, did not consume any prohibited medications, and had evaluable data as specified in the protocol. The detailed study participant disposition and demographic distribution are presented in Table 1 and Supplementary Table 2.

**TABLE 1:** Primary outcome of GSRS questionnaire.

	Visits	Categories	beLP1^®^ (*N* = 49)	Placebo (*N* = 54)	#*P*-value vs. placebo	*P-value vs. baseline
GSRS total score	Day 0	Mean (SD)	30.98 (5.45)	29.61 (4.13)	0.1520^@^	–
	Day 42	Mean (SD)	25.94 (7.80)	26.48 (5.67)	0.0215	beLP1^®^: (0.0001) Placebo: (0.0001)
	Day 84	Mean (SD)	21.22 (10.30)	22.89 (7.21)	0.0394	beLP1^®^: (0.0001) Placebo: (0.0001)
Abdominal pain score	Day 0	Mean (SD)	6.78 (1.91)	6.61 (1.98)	0.6690^@^	–
	Day 42	Mean (SD)	5.73 (2.16)	6.17 (1.81)	0.0235	beLP1: (<0.0001) Placebo: (0.0076)
	Day 84	Mean (SD)	4.65 (2.59)	5.39 (1.75)	0.0205	beLP1: (<0.0001) Placebo: (<0.0001)
Bowel dysfunction syndrome score	Day 0	Mean (SD)	9.59 (2.23)	9.22 (1.86)	0.3611^@^	–
	Day 42	Mean (SD)	8.06 (2.78)	8.20 (2.54)	0.1563	beLP1: (<0.0001) Placebo: (<0.0001)
	Day 84	Mean (SD)	6.41 (3.72)	6.72 (3.18)	0.1995	beLP1: (<0.0001) Placebo: (<0.0001)
Dyspeptic syndrome score	Day 0	Mean (SD)	9.98 (1.89)	9.67 (1.63)	0.3682^@^	–
	Day 42	Mean (SD)	7.69 (3.34)	8.19 (2.12)	0.0721	beLP1: (<0.0001) Placebo: (<0.0001)
	Day 84	Mean (SD)	6.12 (3.71)	7.20 (2.60)	0.0303	beLP1: (<0.0001) Placebo: (<0.0001)
Indigestion syndrome score	Day 0	Mean (SD)	6.82 (1.84)	6.28 (1.70)	0.1260^@^	–
	Day 42	Mean (SD)	6.04 (1.74)	5.67 (1.88)	0.8225	beLP1: (0.0002) Placebo: (0.0002)
	Day 84	Mean (SD)	5.24 (2.44)	5.06 (1.97)	0.5639	beLP1: (<0.0001) Placebo: (<0.0001)

N, number of participants, SD; standard deviation. *p-values were calculated using paired *t*-test. #*p*-values were calculated by using ANCOVA with treatment as factor and baseline value as covariate. @*p*-values were calculated using Students *t*-test.

### 3.2 Participants reported outcomes: GI symptoms (GSRS), perceived stress (PSS), and digestive QoL (DQLQ)

After 84 days of intervention, significant differences were observed in the primary outcome (total GSRS scores) as well as the secondary outcomes (PSS and DQLQ scores) compared to baseline (Figure 2 and Table 1). By Day 42, participants receiving beLP1^®^ demonstrated significantly greater improvements than placebo across multiple questionnaire-based outcomes. Total GSRS scores showed a significantly larger decrease in the beLP1^®^ group (*p* = 0.0215) compared to placebo. This effect was sustained through Day 84, with a lower mean GSRS score from baseline in the beLP1^®^ group (21.22 [10.30]) compared with placebo (22.89 [7.21]), corresponding to a considerably larger improvement in gastrointestinal symptom severity (*p* = 0.0394). Consistent with the reductions observed in total GSRS scores, domain-level analyses revealed broader gastrointestinal benefits with beLP1^®^ supplementation. Significant improvements in abdominal pain were evident in the beLP1^®^ group at both Day 42 and Day 84 compared with placebo (*p* = 0.0235 and *p* = 0.0205, respectively), indicating effective and sustained pain relief. In the dyspeptic syndrome domain, beLP1^®^ produced a significant improvement by Day 84 (*p* = 0.0303). Although between-group differences in bowel dysfunction and indigestion scores did not reach statistical significance, consistent numerical advantages in favor of beLP1^®^ were observed across both domains at both time points, with a notable favorable trend in indigestion symptoms (Table 1). The distribution of individual-level responses across all GSRS subscales is presented in Figure 1, providing granular insight into the breadth and clinical relevance of these symptomatic improvements.

**FIGURE 2:**
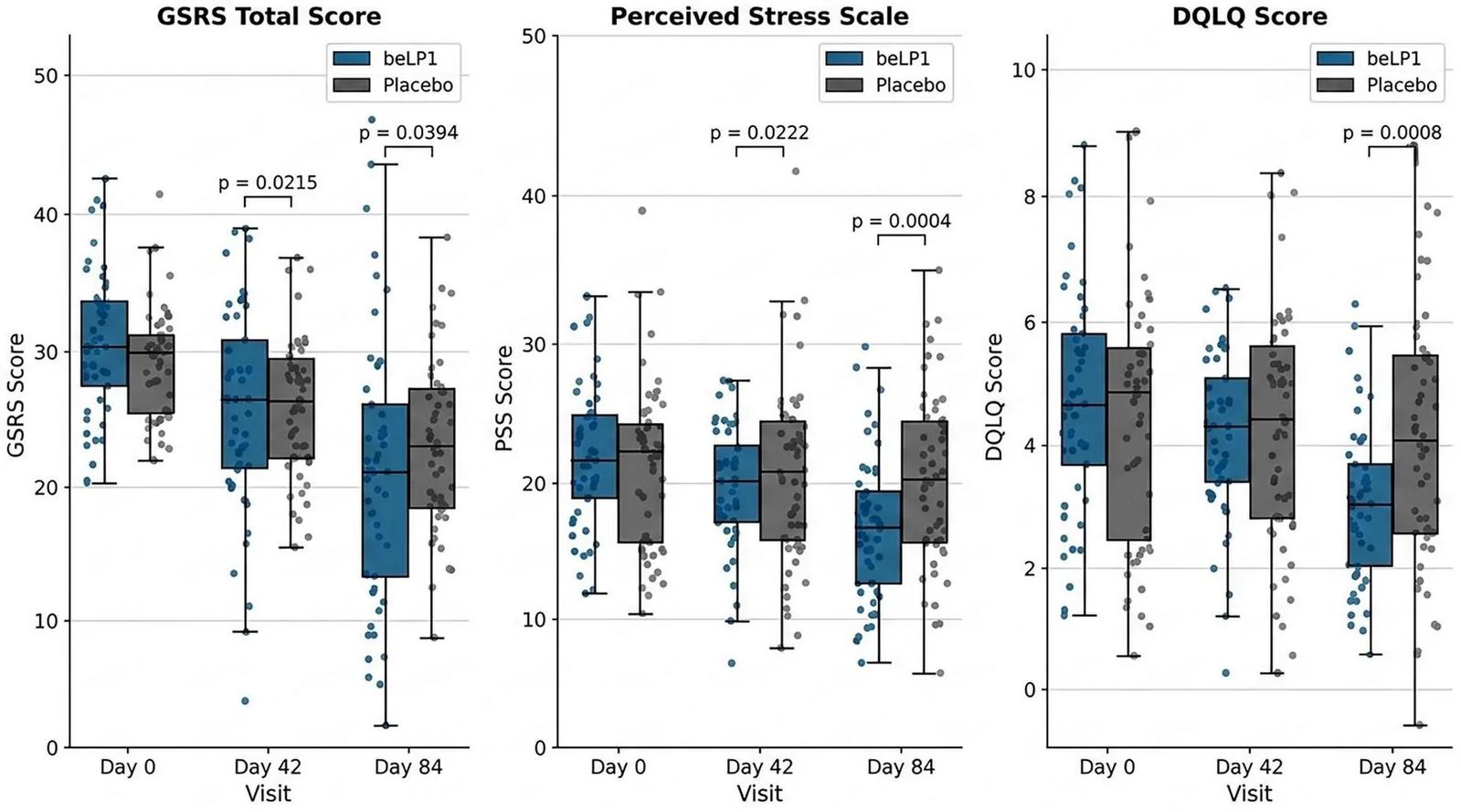
Changes in gastrointestinal symptoms, perceived stress, and quality of life index metrics throughout the intervention. Evaluation of the Gastrointestinal Symptom Rating Scale score trends across groups. Tracking of the longitudinal psychological stress severity index metrics. Assessment profiles for the specific disease-related quality of life questionnaire indices. Values are expressed as means ± SD.

Perceived Stress Scale scores decreased over time in both groups; however, the reduction produced by beLP1^®^ was significantly greater with beLP1^®^ from baseline to Day 42 (*p* = 0.0001) and compared with placebo (*p* = 0.0222) (Figure 2). This effect persisted through Day 84, with a larger decrease in PSS scores in the beLP1^®^ group [16.29 (5.26)] than in the placebo group ([19.44 (7.02)]), resulting in a significantly greater reduction (*p* = 0.0004) (Figure 2).

Similarly, DQLQ score improved significantly from baseline at Day 42 with beLP1^®^ (*p* = 0.0050), with minimal change in the placebo group. By Day 84, beLP1^®^ led to a highly significant improvement in DQLQ scores (*p* < 0.0001), whereas the placebo group showed a smaller but significant decrease (*p* = 0.0022); the magnitude of improvement with beLP1^®^ was greater, evidenced by a significant between-group difference (*p* = 0.0008) (Figure 2). Overall, these findings indicate that beLP1^®^ supplementation yields sustained and statistically significant improvements in gastrointestinal symptoms, perceived stress, and digestion-related quality of life over the intervention period (Figure 2).

### 3.3 Effects of the beLP1^®^ on TNF- α

A decrease in TNF-α levels was observed in the beLP1^®^ group on both Day 42 and Day 84; conversely, the placebo group showed no notable decrease on Day 42, followed by a minimal decrease on Day 84 (Supplementary Table 3). By the end of the study, these differences did not reach statistical significance between groups (*p* = 0.6869), indicating no significant differences between the beLP1^®^ and placebo group (Supplementary Table 3).

### 3.4 Effects of beLP1^®^ on body composition

In the beLP1^®^ group, a significant reduction from baseline in total body fat percentage was observed at Day 42 (*p* = 0.0180), but this effect was not maintained on Day 84. Significant reductions in android (*p* = 0.0437) and gynoid fat percentages (*p* = 0.0020) were also noted at Day 42, while a reduction in gynoid fat percentage was also observed in the placebo group (*p* = 0.0180). No significant changes were detected in lean body mass, fat-free mass, or the android-to-gynoid fat ratio at any assessment (Supplementary Table 4).

### 3.5 Effects of beLP1^®^ on lipid profile

Throughout the study, only minimal changes from baseline were observed in total cholesterol and triglyceride levels in both groups, with values remaining generally stable through Day 84 and no significant between-group differences detected. LDL and HDL cholesterol levels showed no notable changes over time in either group, and no statistically significant within- or between-group differences were observed at any assessment. Overall, beLP1^®^ supplementation did not result in statistically significant changes in lipid profile parameters compared with placebo during the study period (Supplementary Figure 3).

### 3.6 Effect of beLP1^®^ on gut microbiome diversity

To explore the potential microbial mechanisms underlying the observed improvements in gastrointestinal symptoms and digestion-related quality of life, overall gut microbiota diversity was evaluated.

Alpha diversity of the gut microbiome was assessed using observed taxa, Shannon index, Simpson index, and Pielou’s evenness at baseline (Day 0) and Day 84 (Supplementary Figure 1). Across all evaluated metrics, no statistically significant differences were observed either within groups over time or between groups at comparable time points (all *p* > 0.05). The number of observed taxa remained comparable between baseline and Day 84 in both cohorts, indicating that overall microbial richness was not substantially altered by the intervention. Similarly, stability in the Shannon and Simpson indices suggested that global microbial diversity and dominance patterns were preserved throughout the study period, while consistent Pielou’s evenness values reflected a stable distribution of microbial taxa (Supplementary Figure 1). Beta diversity was subsequently evaluated using Principal Coordinates Analysis (PCoA) to characterize differences in global gut microbial community structure between the placebo and beLP1^®^ groups. The PCoA plot demonstrated substantial spatial overlap between samples from both cohorts (Supplementary Figure 2). Statistical testing via pseudo-permutational multivariate analysis of variance (pseudo-PERMANOVA) confirmed no significant differences in microbial community structure between the groups at Day 84 (*p* = 0.4820 and *p* = 0.3500). Taken together, these findings indicate that while beLP1^®^ supplementation maintains a stable ecosystem balance, it does not induce disruptive shifts in global gut microbiome diversity (Supplementary Figures 1, 2).

### 3.7 Modulation of gut microbiome taxonomic composition and profiles

At day 84, the relative abundance of the Bacteroidota phylum, commonly associated with SCFA production, remained largely unchanged across groups (Figure 3). In contrast, the beLP1^®^ group exhibited notable modulation of the Bacillota (formerly Firmicutes) and Pseudomonadota phyla. Bacillota, a Gram-positive phylum comprising several key SCFA-producing taxa (27), showed changes consistent with increased abundance of beneficial fermentative genera observed at lower taxonomic levels. This suggests a potential enhancement in microbial fermentation capacity and in SCFA production, both of which are critical for maintaining gut metabolic homeostasis (27). Notably, changes in the Pseudomonadota (also referred to as Proteobacteria), a major Gram-negative and one of the dominant phyla in the gut microbiome, are particularly relevant. This phylum includes both commensal and potentially pathogenic genera such as *Salmonella* and *Shigella*, and its relative abundance is often considered a marker of microbial imbalance. A reduction or controlled modulation of Proteobacteria abundance is generally considered favorable, as overrepresentation has been associated with gut barrier dysfunction, inflammation, and dysregulated immune responses (28). Thus, the observed modulation in this phylum may indicate improved microbial balance and potential support of gut barrier integrity and immune homeostasis (Figures 3A,B).

**FIGURE 3:**
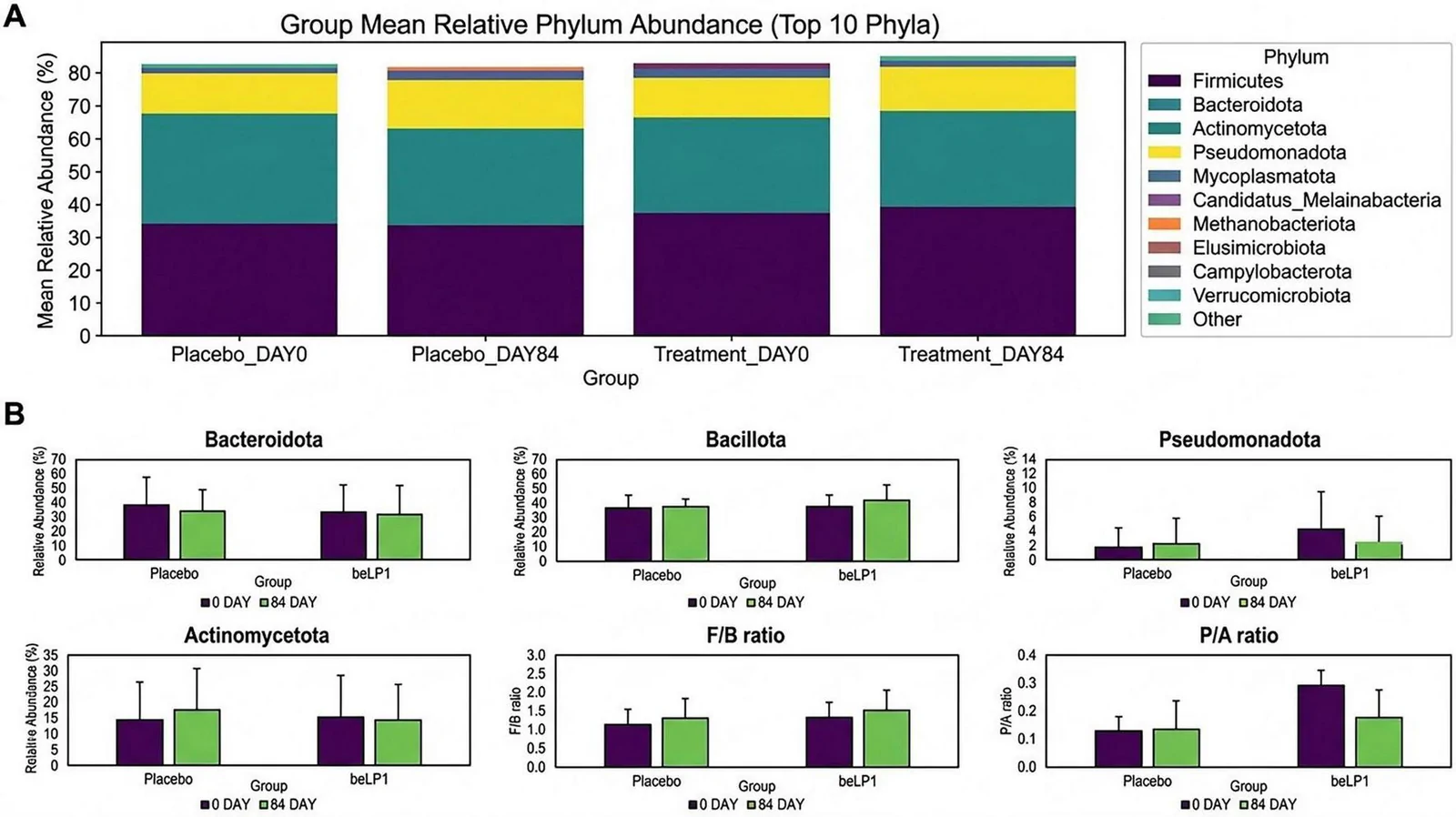
Taxonomic composition and relative abundance shifts of the gut microbiota at the phylum level. **(A)** Top 10 phylum. **(B)** Fecal microbial profiles are summarized at baseline (Day 0) and study endpoint (Day 84) across both the Placebo group (*n* = 54) and the active formula group (beLP1, *n* = 49). Values are expressed as means ± SD.

By day 84, beLP1^®^ supplementation was associated with increased abundance of beneficial SCFA-producing genera, including *Megamonas*, *Blautia*, *Lactobacillus*, and *Agathobacter*. In parallel, a marked reduction was observed in opportunistic genera such as *Escherichia*, previously reported to be associated with intestinal infections and *Klebsiella*, which may exhibit pathogenicity depending on species-specific virulence factors, both of which showed significant decreases (*p* < 0.0001) (Figures 4A–C).

**FIGURE 4:**
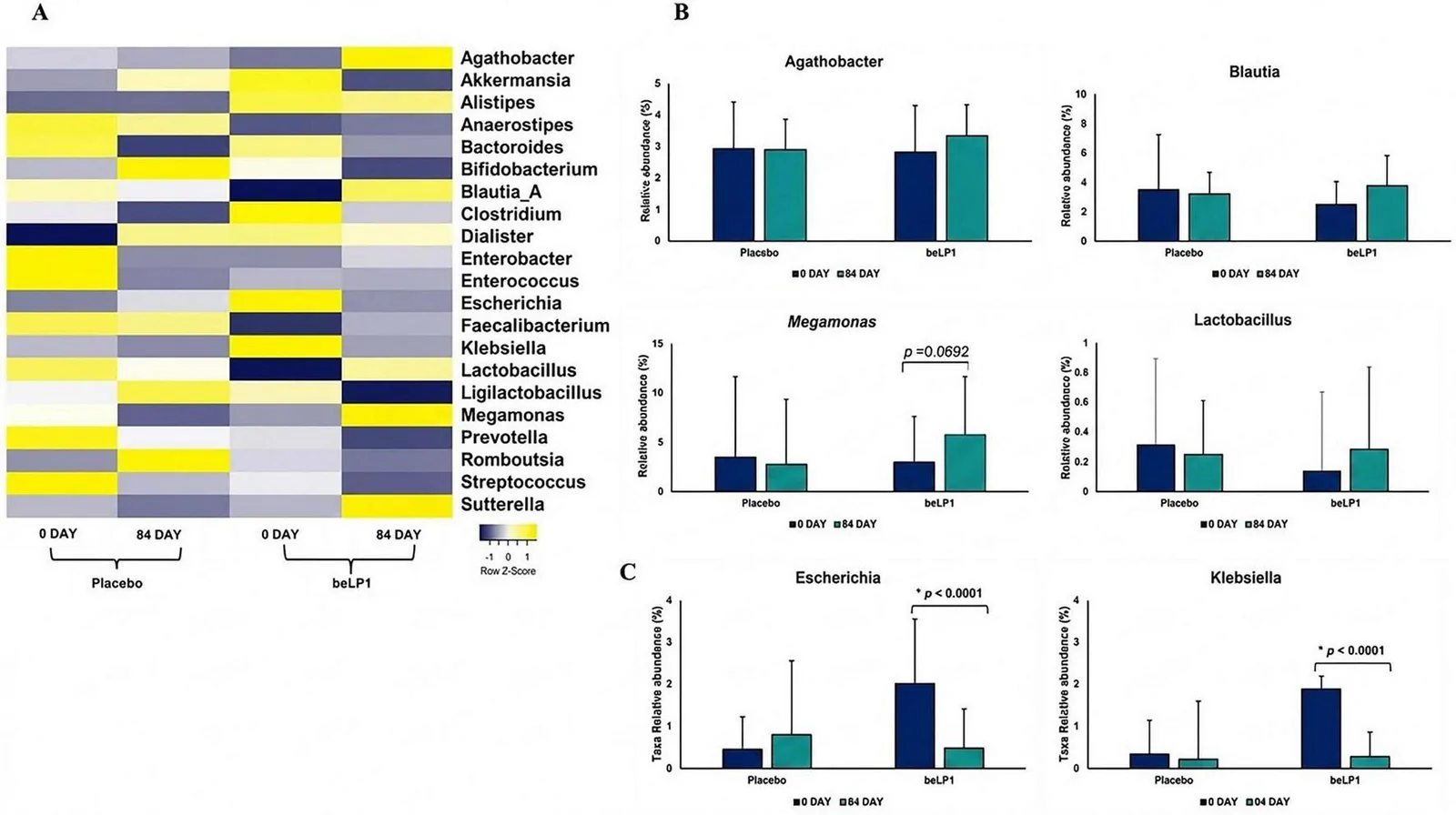
Alterations in gut microbial composition at the genus level following intervention. High-resolution taxonomic profiling was conducted at baseline (Day 0) and study endpoint (Day 84) across the experimental cohorts. **(A)** Heatmap representing top 20 species: Relative abundance changes shift along a blue (low abundance, Z ≤ –1) to red (high abundance, Z ≥ 2) diverging color spectrum. **(B)** Genus-level analysis of beneficial gut bacteria. **(C)** Genus-level analysis of opportunistic bacteria. Significant differences over time and between groups are shown with exact *p*-values calculated via two-tailed tests. Values are expressed as means ± SD.

On day 84, significant enrichment of beneficial species of *Megamonas funiformis* (*p* < 0.0001) was noted, including a remarkable increase in several other beneficial species of *Faecalibacterium longum, Bifidobacterium longum*, *Blautia wexlerae*, and *Blautia* (unclassified). Concurrently, opportunistic pathogens such as *Escherichia coli* and *Klebsiella pneumoniae* were significantly reduced (*p* = 0.0022 and *p* = 0.0309, respectively), reflecting a favorable shift in microbial composition (Figures 5A–C and Supplementary Figure 4).

**FIGURE 5:**
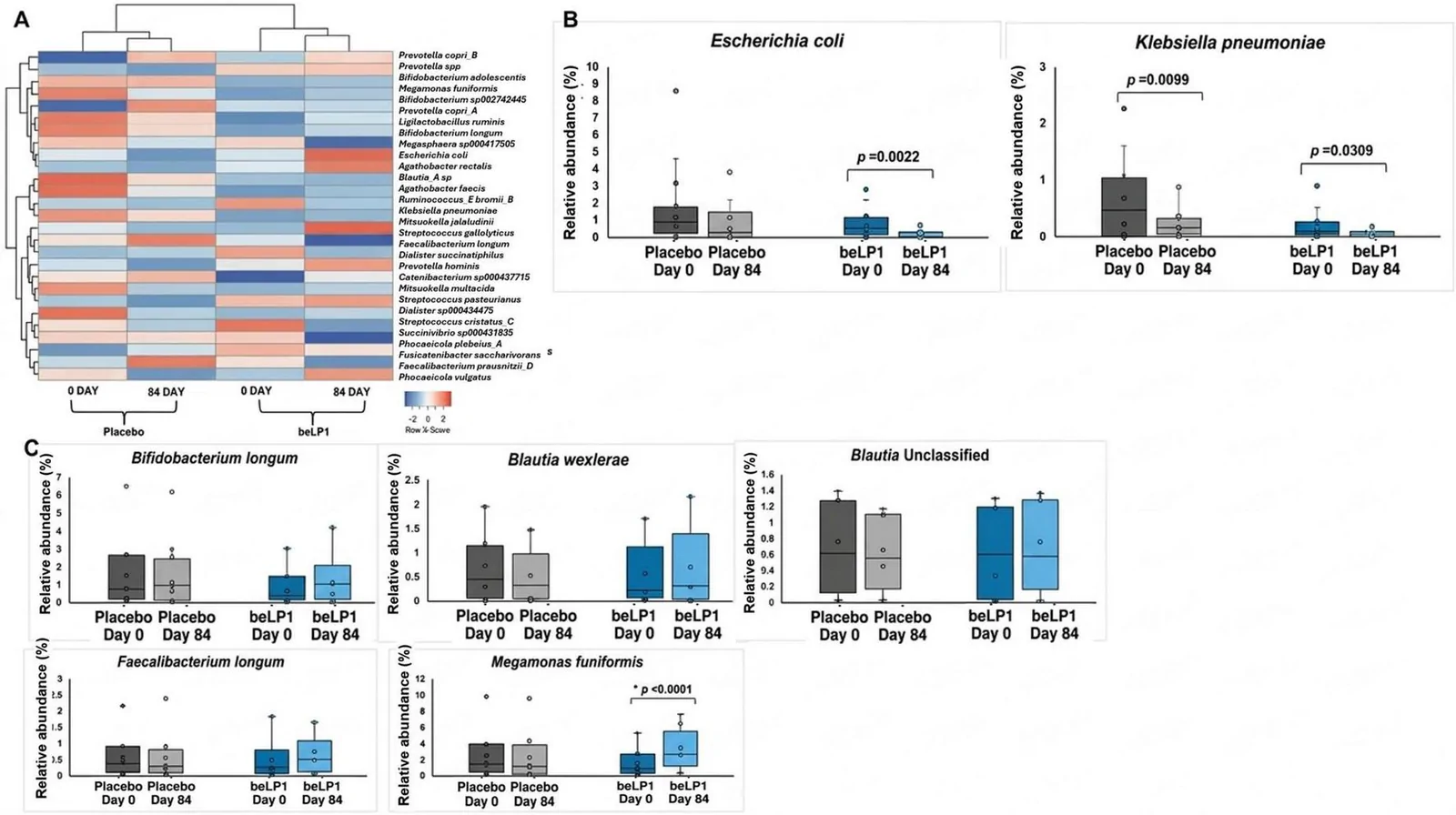
Alterations in gut microbial composition at the species level following intervention. **(A)** Heatmap representing top 30 species **(B)** Opportunistic bacteria **(C)** Beneficial gut bacteria. Values are expressed as means ± SD.

Analysis of antibiotic resistance genes showed a slight, non-significant reduction in overall ARG abundance in the beLP1^®^ group at day 84. Relative abundances across carbapenem, vancomycin, cephalosporin, and extended-spectrum β-lactamase (ESBL) categories remained stable throughout the study, with no meaningful differences observed between baseline and day 84 or between groups, indicating that beLP1^®^ supplementation did not alter the gut antibiotic resistance profile (Figure 6 and Supplementary Figure 5).

**FIGURE 6:**
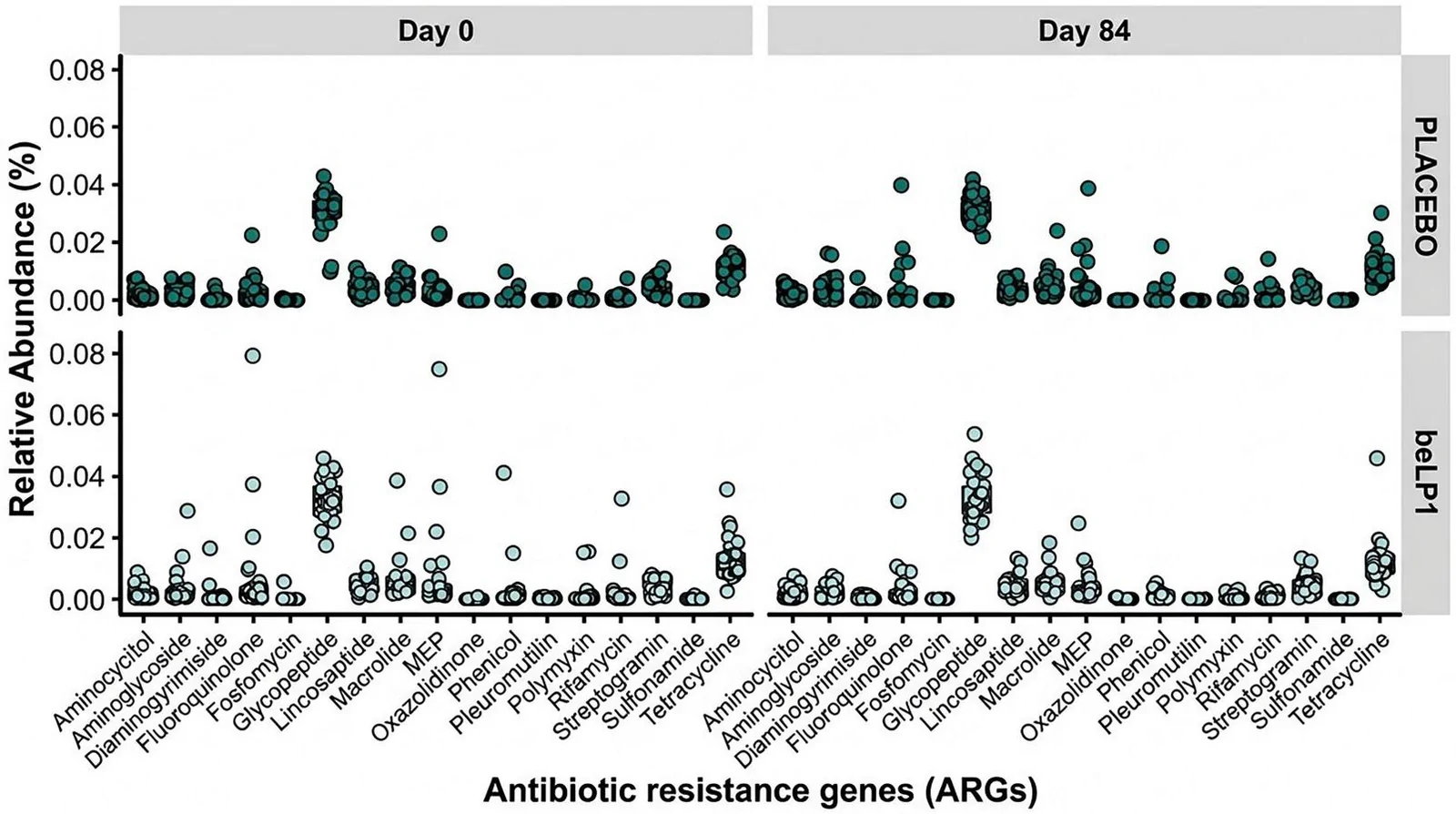
Alterations and profiling of gut metagenomic antibiotic resistance genes (ARGs) following intervention. Metagenomic resistome mapping was conducted at baseline (Day 0) and study endpoint (Day 84) across the experimental cohorts.

### 3.8 Effect of the beLP1^®^ administration on safety parameters and adverse event outcomes

Vital signs, including pulse rate and systolic and diastolic blood pressure, were assessed at baseline and planned follow-up visits throughout the 84-day study period, with no significant changes observed in either group. During the 84-day intervention period, a total of 25 adverse events were reported across both study groups: 12 in the beLP1^®^ group and 13 in the placebo group. Of these, two were classified as moderately severe; the remainder were mild and were not considered related to the investigational postbiotic product. All events resolved completely, with no consequences or sequelae (Supplementary Table 5).

## 4 Discussion

Gut homeostasis is fundamental to human health, with accumulating evidence underscoring the pivotal role of the gut microbiota in sustaining physiological equilibrium. Host–microbe interactions regulate digestive processes, immune responses, aging, and vulnerability to disease (29). The gut microbiota has been recognized as a key contributor to the development and progression of obesity and its associated metabolic disorders (30). This underscores the need for strategies to restore gut microbiota balance and maintain a healthy intestinal environment, thereby reducing the risk of related diseases. Accordingly, this study investigated the effects of a new postbiotic strain, heat-killed beLP1^®^, on gut health in individuals with gastrointestinal symptoms.

Growing evidence indicates that postbiotics can directly support gastrointestinal health by modulating inflammatory responses, strengthening gut barrier function, and potentially exerting synergistic effects when combined with other therapeutic agents (31). In line with improvements in gastrointestinal symptoms, Naghibi et al. reported a 19.05% reduction in total GSRS scores after 10 weeks of supplementation with *Bifidobacterium longum* CECT 7347 (32). Consistent with this, the study’s primary outcome showed that beLP1^®^ supplementation resulted in significantly greater reductions in total GSRS scores than placebo at Day 42 and Day 84. Although the primary per-protocol sample size of 103 participants (*n* = 49 in beLP1^®^ and *n* = 54 in placebo) provided 80% statistical power to detect clinically meaningful reductions in GSRS total scores, larger multi-center cohorts with extended follow-up durations are recommended to evaluate long-term ecological and metabolic outcomes. These reductions corresponded to 16.26% and 31.50% decreases on Day 42 and Day 84, respectively, indicating a progressive improvement in gastrointestinal symptoms over time. These improvements compare favorably with clinical outcomes observed in trials using live *L. plantarum* strains over an identical 84-day timeframe, which similarly demonstrated significant symptom relief in abdominal pain and dyspeptic discomfort (12). Domain-wise analysis demonstrated significant improvements in abdominal pain with beLP1^®^ at Day 42 (*p* = 0.0235) and Day 84 (*p* = 0.0205), and a significantly greater reduction in dyspeptic symptoms by Day 84 (*p* = 0.0303). This indicates the beneficial role of beLP1^®^ in alleviating abdominal pain and dyspeptic syndrome.

Findings from Luppino et al.’s meta-analysis highlight a bidirectional relationship between obesity and depression, emphasizing that each condition acts as a risk factor for the development of the other. Singh RG et al. reported that after 84 days of probiotic supplementation with *L. plantarum* strains, more than 50% of participants transitioned from moderate stress (PSS > 13 to ≤26) to low stress levels (PSS ≤ 13) (12, 33). The observed reduction in perceived stress was approximately threefold greater in the beLP1^®^ group than in the placebo group (*p* = 0.0004). Considering this substantial absolute change, the between-group difference highlights a potential role for beLP1^®^ in supporting host stress resilience, likely mediated through neuro-immune and neuro-endocrine signaling pathways along the gut-brain axis (34). Given that psychological stress directly exacerbates visceral hypersensitivity and enteric motor dysfunction, this reduction in stress likely contributed synergistically to the observed alleviation of physical gastrointestinal symptoms (33).

Evidence suggests that excess body weight is associated with poorer gastrointestinal-related quality of life (35). In the current analysis, beLP1^®^ supplementation significantly improved (*p* = 0.0008) digestion-related quality of life, as reflected by reductions in DQLQ scores at Day 42 and a sustained, greater effect by Day 84. These improvements are consistent with the significant reductions observed in key GSRS domains, particularly abdominal pain and dyspeptic syndrome, which are known to substantially affect patient’s perceived digestive well-being. The alignment between improvements in these symptom-specific domains and overall DQLQ scores supports the clinical relevance of the observed quality-of-life benefits.

Furthermore, while TNF-α is critical for maintaining gut homeostatic balance through cell turnover, its dysregulation drives the inflammatory cascade observed in GI disorders (36). Moreover, obesity-associated gut microbiota dysbiosis has been shown to promote intestinal inflammation and increase pro-inflammatory cytokine production, including TNF-α, which, in turn, influences epithelial turnover and barrier integrity, thereby contributing to gut homeostasis (37). In the present study, serum TNF-α levels showed numerical reductions in the beLP1^®^ group but did not achieve statistical significance compared to placebo (*p* = 0.6869). This lack of marked systemic cytokine modulation indicates that the clinical benefits of beLP1^®^ are primarily driven through localized mucosal interactions within the gastrointestinal tract rather than broad systemic immune suppression (8, 36). In cohorts of otherwise healthy adults with mild-to-moderate GI symptoms, baseline inflammatory cascades are typically low, leaving limited headroom for significant systemic biomarker reduction without concurrent systemic challenge (37).

Interactions among the gut microbiota, host genetics, diet, and environmental factors are well known to influence lipid metabolism and metabolic health (38). In the present study, metabolic parameters remained largely stable across the intervention period: total cholesterol showed only a modest numerical decrease with beLP1^®^, triglycerides declined similarly in both groups, and LDL and HDL cholesterol levels remained unchanged. Although several studies evaluating live *L. plantarum* strains have reported anti-adipogenic or lipid-lowering efficacy, our results align with clinical trials demonstrating that postbiotics frequently exert strain-specific, localized gastrointestinal responses rather than systemic metabolic changes (11, 39). For instance, Lee et al. reported that 6 weeks of heat-killed *L. plantarum TWK10* supplementation effectively reduced fatigue and modulated gut microbiota in humans without producing pronounced changes in body composition (11). The absence of significant lipid or DEXA body composition shifts in our 84-day trial may reflect the inanimate nature of heat-killed cells which act via mucosal cell-wall interaction (e.g., TLR2 signaling) rather than continuous *in situ* metabolite production as well as the absence of a concurrent dietary or caloric restriction protocol (6, 7).

The gut microbiome is a complex ecosystem that plays a vital role in host health by supporting nutrient and mineral absorption, fermenting dietary fibers into SCFAs, synthesizing vitamins, detoxifying harmful compounds, and regulating immune function (40). In the present study, the beLP1^®^ group exhibited notable modulation at the phylum level, particularly within Bacillota (formerly Firmicutes) and Pseudomonadota, suggesting a shift toward a more favorable microbial composition. In line with this beLP1^®^ intervention significantly increased relative abundance of beneficial bacterial species, including *Bifidobacterium adolescentis*, *Segatella copri*, *Megamonas funiformis*, *Faecalibacterium prausnitzii, Roseburia faecis, Blautia wexlerae, Phocaeicola vulgatus*, and to some extent *Agathobacter rectalis*. The observed increase in *Faecalibacterium longum* aligns with clinical improvements in gastrointestinal symptoms and quality-of-life measures in this study. This genus has been associated with a healthier intestinal environment (41), whereas decreased levels have been linked to metabolic and gastrointestinal disorders (42).

In the present study, 84 days of beLP1^®^ supplementation did not significantly alter global gut microbiota architecture, as demonstrated by stable alpha diversity indices (observed taxa, Shannon, Simpson, Pielou’s evenness) and overlapping beta diversity profiles. Rather than indicating a lack of efficacy, the preservation of baseline diversity reflects the inherent ecological resilience of the adult gut microbiome during short-term interventions (43, 44). Indeed, systematic reviews and clinical trials consistently demonstrate that effective microbiome interventions in adult populations frequently drive targeted species-level adjustments while maintaining overall community homeostasis (45, 46).

Also, during the 84-day intervention period with beLP1^®^, relative abundance of *Megamonas funiformis* increased significantly (*p* < 0.0001), and it has been reported to be associated with improved gut health and modulation of the gut microbiota in the context of metabolic dysfunction–associated fatty liver disease (44). Alongside enrichment of *Bifidobacterium longum*, *Blautia wexlerae*, and *Blautia* (unclassified); *Escherichia coli* and *Klebsiella pneumoniae* were significantly reduced (*p* = 0.0022 and *p* = 0.0309), indicating a beneficial shift in gut microbiota composition. This aligns with mechanistic and observational data indicating that limiting the abundance of these two harmful species may be associated with reduced energy harvesting, systemic inflammation, and adiposity, thereby improved metabolic health (47). These findings suggest that beLP1^®^ may promote gut health by partially normalizing microbial profiles typically disrupted in obesity, type 2 diabetes, and intestinal inflammation. *Bifidobacterium adolescentis* is known to promote gastrointestinal health by strengthening the gut barrier, modulating inflammatory responses, improving metabolic function, and producing beneficial metabolites such as acetate and lactate (48). Similarly, *Megamonas funiformis* has been reported to improve gut health by modulating metabolic dysfunction–associated fatty liver disease through alterations in gut microbial composition (45). After 84 days of beLP1^®^ administration, several beneficial bacteria, such as: *Bifidobacterium*, *Faecalibacterium longum* and *Agathobacter rectalis* increased.

Moreover, the lack of meaningful change in the gut ARG profile over 84 days suggests that beLP1^®^ does not substantially expand the gut antibiotic resistance gene pool, aligning with the literature indicating that probiotic effects on ARGs are often minimal or person-specific rather than broadly augmenting resistance determinants in the microbiome (49). Overall, these microbiome changes suggest that beLP1^®^ positively modulates gut microbial composition by increasing beneficial bacteria and decreasing potentially harmful species, supporting better gastrointestinal and metabolic health. From a safety perspective, beLP1^®^ supplementation was generally well tolerated, with no serious adverse events reported and a comparable incidence of adverse events across the study groups, most of which were mild and resolved completely.

While this study provides robust evidence for the efficacy of beLP1^®^, several limitations should be considered. Although the sample size and 84-day duration were statistically sufficient to demonstrate meaningful clinical and taxonomic changes, they may not have been extensive enough to capture subtle systemic biomarker shifts or long-term ecological trajectories. Additionally, because this cohort comprised individuals experiencing functional gastrointestinal symptoms rather than diagnosed organic diseases, these findings specifically highlight the utility of beLP1^®^ for restoring gastrointestinal comfort and managing subclinical dysbiosis in the general population. Furthermore, by restricting the trial population to adults aged 18–45 years to minimize age-related physiological and microbiome-associated confounders, the generalizability of these findings to older populations remains to be established. Future clinical investigations should evaluate beLP1^®^, in older demographics experiencing gastrointestinal and metabolic dysbiosis. Change in diet and physical activity are known to influence gut microbiota composition and gastrointestinal health. Participants were instructed to maintain their usual dietary and physical activity habits throughout the study; however, these factors were not quantitatively monitored and therefore cannot be completely excluded as potential confounders. Nevertheless, the high consistency observed across symptom relief, quality-of-life improvements, and favorable microbiome modulation underscores the clinical promise of beLP1^®^ and strongly supports its further validation in larger, long-term multi-center cohorts.

## 5 Conclusion

Overall, our findings demonstrate that beLP1^®^ supplementation provides rapid and progressive benefits for gastrointestinal health, with significant improvements emerging at Day 42 and microbially consolidating by Day 84. These clinical improvements were characterized by marked reductions in global gastrointestinal symptoms and localized domains specifically abdominal pain and dyspeptic syndrome as well as enhanced digestion-related quality of life and reduced perceived stress. Mechanistically, these symptom reductions occurred in parallel with a targeted optimization of the gut microbiota taxonomic profile. The intervention successfully enriched key health-associated commensals, including *B. longum*, *F. prausnitzii*, *Blautia* spp., and *M. funiformis*, while simultaneously suppressing the opportunistic pathogens *E. coli* and *K. pneumoniae*. In contrast, systemic inflammatory markers, including TNF-α remained stable, indicating that the clinical efficacy of beLP1^®^ is primarily driven through localized gastrointestinal and host-microbiome interactions rather than systemic immune modulation. Furthermore, the stability of the colonic resistome confirms the ecological safety of this intervention. In conclusion, daily supplementation with the heat-killed postbiotic beLP1^®^ over 84 days safely and progressively reduced gastrointestinal symptoms, perceived stress, and quality of life impairment in a per-protocol cohort of 103 adults with excess weight (*n* = 49 vs. *n* = 54 placebo).

## Data Availability

The datasets generated and/or analysed during the current study are available in the NCBI Sequence Read Archive (SRA) repository under BioProject accession number PRJNA1493290.
