# Supplementary material for: Gut health and microbiome modulation by the heat-killed postbiotic beLP1® in adults with excess weight: a randomized, placebo-controlled trial
Source: Front Nutr. 2026 Sep 17;13:1919277. doi: 10.3389/fnut.2026.1919277 (PMC13627065; doi:10.3389/fnut.2026.1919277)
Supplement: Supplementary file 1 [file Table_1.docx]

**Gut health and microbiome modulation by the heat-killed postbiotic beLP1^®^ in adults with excess weight: a randomized, placebo-controlled trial**

Ranjith Kumar Manoharan^1†^, Kwon Il Han^1^, Yura Lee^1^, Sunhwa Baek^1^, Eunjung Moon^1^, Youn Bum Park^1^, Junhui Cho^1^, Jayesh Chaudhary^2^, ^1^, Hyun-Dong Shin^1†^

^1^Research & Development Center, Bereum Co., Ltd., Wonju 26361, Republic of Korea

^2^Vedic Lifesciences, 203, Morya Landmark 1, Off New Link Road, Andheri West 400053, Mumbai, India

**Table S1. Eligibility criteria for participation in the study.**

**Inclusion criteria**

1. BMI between 25 to 35 kg/m^2^ (both values included).
2. Individuals with liver & renal function test values as defined below:

- Individuals with ALT, AST values ≤ 2 times of the upper limit of normal (ULN).
- Individuals with creatinine values ≤ 1.5 times of the upper limit of normal (ULN).
- Individuals with ALP values between 38 and 126 U/L (both values included).

3. Had two of the following five metabolic risk factors:
   i. Waist circumference greater than 102 cm (40 inches) for men and greater than 88 cm (35 inches) for women;
   ii. Fasting triglycerides greater than 150 mg/dl and less than 300 mg/dl.
   iii. Blood pressure greater than or equal to 130 mm Hg SBP (Systolic Blood Pressure) and/or greater than or equal to 85 mm Hg DBP (Diastolic Blood Pressure).
   iv. Fasting blood glucose (FBG) greater than or equal to 100 mg/ dl.
   v. Fasting HDL cholesterol level less than or equal to 40 mg/dl (men) or less than or equal to 50 mg/dl (women).

**Exclusion criteria**

1. Individuals with unstable, symptomatic, or clinically significant medical conditions, including major renal, hepatic, endocrine, gastrointestinal, pancreatic, neurologic, or psychiatric disorders.
2. Individuals with diagnosed diabetes or fasting blood glucose >125 mg/dL; those with hypertension requiring medication or uncontrolled blood pressure (systolic ≥150 mmHg or diastolic ≥100 mmHg); or those with thyroid dysfunction based on TSH levels outside 0.4–4.3 mIU/L.
3. Individuals who regularly smoke or use tobacco, or who consume ≥3 servings of alcohol per day.
4. Individuals who regularly consume probiotic supplements and are unwilling to stop at least one week prior to screening and throughout the study. Supplemental probiotics may include standalone probiotic supplements, vitamins with probiotics, and any foods supplemented with probiotics.
5. Pregnant or lactating women, those planning pregnancy, or those with known allergies or intolerances to study product ingredients.

Individuals unable or unwilling to comply with study procedures, deemed unsuitable by the investigator, or currently participating in another clinical trial.

**Table S2. Demographics and Baseline Characteristics (Safety population)**

| **Parameter** | **Statistics** | beLP1^®^ **(N=70)** | **Placebo (N=70)** | **Overall (N=140)** | **P-value** |
| --- | --- | --- | --- | --- | --- |
| Age (years) | Mean (SD) | 34.94(6.60) | 34.44(6.93) | 34.69(6.75) | 0.6626 (T) |
|  | Min, Max | 20.00, 45.00 | 21.00, 45.00 | 20.00, 45.00 |  |
| Gender n (%) | Male | 28 (40.00%) | 33 (47.14%) | 61 (43.57%) | 0.3941(C) |
|  | Female | 42 (60.00%) | 37 (52.86%) | 79 (56.43%) |  |
| Height (Meters) | Mean (SD) | 1.62(0.10) | 1.62(0.10) | 1.62(0.10) | 0.9408 (T) |
|  | Min, Max | 1.41, 1.85 | 1.42, 1.86 | 1.41, 1.86 |  |
| Weight (Kg) | Mean (SD) | 78.13(12.53) | 77.46(11.45) | 77.79(11.96) | 0.7430 (T) |
|  | Min, Max | 54.10, 102.20 | 53.90, 106.60 | 53.90, 106.60 |  |
| BMI (Kg/m^2^) | Mean (SD) | 29.83(3.03) | 29.58(2.95) | 29.70(2.98) | 0.6174 (T) |
|  | Min, Max | 25.15, 34.98 | 25.29, 34.93 | 25.15, 34.98 |  |
| Waist circumference(cms)-(Avg) | Mean (SD) | 103.95(7.99) | 104.12(8.02) | 104.04(7.98) | 0.8974 (T) |
|  | Min, Max | 89.15, 123.00 | 88.80, 126.70 | 88.80, 126.70 |  |
| Waist/Height Ratio | Mean (SD) | 0.64(0.05) | 0.65(0.05) | 0.65(0.05) | 0.9128 (T) |
|  | Min, Max | 0.57, 0.76 | 0.56, 0.78 | 0.56, 0.78 |  |
| Notes [1] p-value was calculated using Student's t-test (T) for continuous variables and Chi Square(C) test for Categorical Variables.  [2] Percentages were calculated using respective column header count as denominator. | | | | | |

**Table S3. Tumor necrosis factor-alpha (PP)**

| **TNF-α levels** | **Categories** | **beLP1^®^ (N= 49)** | **Placebo (N=54)** | **# p-value vs. placebo** | *** p-value vs. baseline** |
| --- | --- | --- | --- | --- | --- |
| Day 0 | Mean (SD) | 0.68 (0.76) | 0.50 (0.34) | 0.1238^@^ | - |
| Day 42 | Mean (SD) | 0.48 (0.20) | 0.50 (0.25) | 0.6156 | beLP1: (0.0807)  Placebo: (0.9005) |
| Day 84 | Mean (SD) | 0.50 (0.27) | 0.48 (0.25) | 0.6869 | beLP1: (0.1204)  Placebo: (0.7153) |
| *Abbreviations: N; Number of participants, SD; Standard deviation*  **p-values were calculated using paired t-test.*  *#p-values were calculated by using ANCOVA with treatment as factor and baseline value as covariate*  *@p-values were calculated using Student’s t-test* | | | | | |

**Table S4. Body composition (DEXA) (PP)**

| **Parameters** | **Visit** | **Categories** | **beLP1^®^ (N= 49)** | **Placebo (N=54)** | **# p-value vs. placebo** | *** p-value vs. baseline** |
| --- | --- | --- | --- | --- | --- | --- |
| Body Fat % | Day 0 | Mean (SD) | 45.60  (7.62) | 43.16  (8.66) | 0.1345^@^ | - |
|  | Day 42 | Mean (SD) | 44.87  (7.89) | 42.84  (8.69) | 0.3493 | beLP1: (0.0180)  Placebo: (0.2179) |
|  | Day 84 | Mean (SD) | 45.14  (8.04) | 42.96  (9.00) | 0.6242 | beLP1: (0.3020)  Placebo: (0.4794) |
| Lean Body Mass (Grams) | Day 0 | Mean (SD) | 39813.37  (8967.62) | 40890.11 (8334.42) | 0.5291^@^ | - |
|  | Day 42 | Mean (SD) | 40113.76  (8677.59) | 41096.31 (8501.97) | 0.8114 | beLP1: (0.1973)  Placebo: (0.2855) |
|  | Day 84 | Mean (SD) | 39821.67  (8799.02) | 41142.72 (8676.72) | 0.4787 | beLP1: (0.9769)  Placebo: (0.2621) |
| Fat Free Mass (Grams) | Day 0 | Mean (SD) | 42209.33  (9271.32) | 43279.09 (8569.51) | 0.5442^@^ | - |
|  | Day 42 | Mean (SD) | 42450.16  (8943.12) | 43434.67  (8717.16) | 0.8349 | beLP1: (0.2931)  Placebo: (0.4050) |
|  | Day 84 | Mean (SD) | 42286.53  (9013.64) | 43125.07  (9126.37) | 0.7242 | beLP1: (0.7845)  Placebo: (0.7418) |
| Android Fat (%) | Day 0 | Mean (SD) | 52.09  (7.33) | 49.67  (8.72) | 0.1331^@^ | - |
|  | Day 42 | Mean (SD) | 51.12  (8.33) | 49.61  (9.79) | 0.1440 | beLP1: (0.0437)  Placebo: (0.9112) |
|  | Day 84 | Mean (SD) | 51.39  (8.70) | 49.75  (9.46) | 0.3552 | beLP1: (0.2570)  Placebo: (0.8870) |
| Gynoid Fat (%) | Day 0 | Mean (SD) | 49.36  (9.19) | 47.03  (9.60) | 0.2127^@^ | - |
|  | Day 42 | Mean (SD) | 48.22  (9.26) | 46.05  (9.84) | 0.8507 | beLP1: (0.0020)  Placebo: (0.0180) |
|  | Day 84 | Mean (SD) | 49.01  (9.45) | 46.75  (9.79) | 0.9671 | beLP1: (0.4302)  Placebo: (0.3849) |
| A/G Ratio | Day 0 | Mean (SD) | 1.07 (0.14) | 1.07 (0.13) | 0.9029^@^ | - |
|  | Day 42 | Mean (SD) | 1.07 (0.12) | 1.09 (0.13) | 0.2019 | beLP1: (0.9256)  Placebo: (0.0416) |
|  | Day 84 | Mean (SD) | 1.06 (0.14) | 1.08 (0.13) | 0.3295 | beLP1: (0.3759)  Placebo: (0.6262) |
| *Abbreviations: CI; Confidence interval, Min; Minimum, Max; Maximum, N; Number of participants, SD; Standard deviation*  **p-values were calculated using paired t-test.*  *#p-values were calculated by using ANCOVA with treatment as factor and baseline value as covariate*  *@p-values were calculated using Student’s t-test* | | | | | | |

**Table S5. Adverse Event (Safety Population)**

| **Adverse Event** | **beLP1^®^ (N=70)** | **Placebo**  **(N=70)** | **Overall (N=140)** |
| --- | --- | --- | --- |
| Bloating | 0 (0.00%) | 1 (1.43%) | 1 (0.71%) |
| Bodyache | 0 (0.00%) | 1 (1.43%) | 1 (0.71%) |
| Burning sensation in abdomen | 1 (1.43%) | 0 (0.00%) | 1 (0.71%) |
| Chest pain | 1 (1.43%) | 0 (0.00%) | 1 (0.71%) |
| Common cold | 0 (0.00%) | 2 (2.86%) | 2 (1.43%) |
| Dry cough | 0 (0.00%) | 1 (1.43%) | 1 (0.71%) |
| Fever | 4 (5.71%) | 1 (1.43%) | 5 (3.57%) |
| Finger injury | 1 (1.43%) | 0 (0.00%) | 1 (0.71%) |
| Headache | 1 (1.43%) | 4 (5.71%) | 5 (3.57%) |
| Heartburn | 2 (2.86%) | 0 (0.00%) | 2 (1.43%) |
| Sneezing | 0 (0.00%) | 1 (1.43%) | 1 (0.71%) |
| Sore Throat | 0 (0.00%) | 1 (1.43%) | 1 (0.71%) |
| Vomiting | 2 (2.86%) | 1 (1.43%) | 3 (2.14%) |
| [1] Percentages were calculated using respective column header count as denominator. | | | |

**Figure S1: Alpha diversity across groups (beLP1^®^ vs Placebo)**

**Figure S2. Beta diversity analysis of gut microbiome using PCoA.**

**Figure S3. Lipid profile parameters compared with placebo during the study period.**

**A**

**B**

**Figure S4: (A) Top Bacterial Species at Day 84 (B) Changes in the Relative Abundance of Key Gut Microbial Species from Day 0 to Day 84.**

**Figure S5. ARGs functional profiling of Placebo (A) and beLP1^®^ (B).**
